# Outer membrane porin M35 of *Moraxella catarrhalis *mediates susceptibility to aminopenicillins

**DOI:** 10.1186/1471-2180-9-188

**Published:** 2009-09-04

**Authors:** Marion Jetter, Nadja Heiniger, Violeta Spaniol, Rolf Troller, André Schaller, Christoph Aebi

**Affiliations:** 1 Institute for Infectious Diseases, University of Bern, CH-3010 Bern, Switzerland; 2 Division of Infectious Diseases, University of California at San Francisco, USA; 3 Division of Human Molecular Genetics, University of Bern, Inselspital, CH-3010 Bern, Switzerland; 4 Department of Pediatrics, University of Bern, Inselspital, CH-3010 Bern, Switzerland

## Abstract

**Background:**

The outer membrane protein M35 is a conserved porin of type 1 strains of the respiratory pathogen *Moraxella catarrhalis*. It was previously shown that M35 is involved in the uptake of essential nutrients required for bacterial growth and for nasal colonization in mice. The aim of this study was (i) to characterize the potential roles of M35 in the host-pathogen interactions considering the known multifunctionality of porins and (ii) to characterize the degree of conservation in the phylogenetic older subpopulation (type 2) of *M. catarrhalis*.

**Results:**

Isogenic *m35 *mutants of the type 1 strains O35E, 300 and 415 were tested for their antimicrobial susceptibility against 15 different agents. Differences in the MIC (Minimum Inhibitory Concentration) between wild-type and mutant strains were found for eight antibiotics. For ampicillin and amoxicillin, we observed a statistically significant 2.5 to 2.9-fold MIC increase (p < 0.03) in the *m35 *mutants. Immunoblot analysis demonstrated that human saliva contains anti-M35 IgA. Wild-type strains and their respective *m35 *mutants were indistinguishable with respect to the phenotypes of autoagglutination, serum resistance, iron acquisition from human lactoferrin, adherence to and invasion of respiratory tract epithelial cells, and proinflammatory stimulation of human monocytes. DNA sequencing of *m35 *from the phylogenetic subpopulation type 2 strain 287 revealed 94.2% and 92.8% identity on the DNA and amino acid levels, respectively, in comparison with type 1 strains.

**Conclusion:**

The increase in MIC for ampicillin and amoxicillin, respectively, in the M35-deficient mutants indicates that this porin affects the outer membrane permeability for aminopenicillins in a clinically relevant manner. The presence of IgA antibodies in healthy human donors indicates that M35 is expressed *in vivo *and recognized as a mucosal antigen by the human host. However, immunoblot analysis of human saliva suggests the possibility of antigenic variation of immunoreactive epitopes, which warrants further analysis before M35 can be considered a potential vaccine candidate.

## Background

*Moraxella catarrhalis *is an exclusively human, mucosal respiratory tract commensal and pathogen causing between 5% [1] and 20% of cases of acute otitis media in children [2] across all regions of the world. The recent introduction of routine infant immunization with pneumococcal conjugate vaccines has - in some studies [3] - led to a substantial increase in otitis media caused by *M. catarrhalis *[3]. It is thus a major cause of the most common bacterial infection in children requiring medical attention. *M. catarrhalis *also triggers approximately 10% acute exacerbations of chronic obstructive pulmonary disease (COPD) in adults [4]

In our attempts to identify cold shock regulated outer membrane proteins (OMP) of *M. catarrhalis *[5] we investigated a recently described OMP called M35. We found no evidence of cold shock regulation, but the construction of an isogenic mutant lacking the expression of a currently incompletely described OMP of *M. catarrhalis *provided us with the opportunity to conduct a phenotypic analysis of the function of M35. Meanwhile, in an elegant series of experiments, Easton and co-workers [6] demonstrated that M35 is a typical Gram-negative OM porin, which also is essential for short-term nasal colonization of mice. Importantly, porins of Gram-negative bacteria not only assure bacterial homeostasis by acting as transport channels, but are also known to afford virulence mechanisms such as adhesion, invasion [7-11], and pro-inflammatory stimulation. [11-17]. In addition, porins are often involved in antimicrobial resistance [18-26]. Porins of *M. catarrhalis *have received little attention in the scientific literature. Gotho et al. described the permeability for beta-lactam antibiotics across the OM of *M. catarrhalis *suggesting that porins may be involved [27]. Lafontaine et al investigated the porin-like OMP CD, which acts as an adhesin on lung cells [7]. Thus, M35 is currently the only well characterized porin of *M. catarrhalis *[6,28].

The aims of the present study were (i) to provide an overview of phenotypic differences between the strains O35E, 300 and 415 and their respective isogenic *m35 *mutants, (ii) to investigate whether M35 is a human mucosal antigen and thus a potential vaccine candidate, (iii) to evaluate the role of M35 in the susceptibility of *M. catarrhalis *to various classes of antimicrobial agents, and (iv) to provide the DNA sequence *m35 *of strain 287, which is a representative of the phylogenetically older major lineage (type 2) of *M. catarrhalis *[29].

## Methods

### Bacterial strains and culture conditions

The *M. catarrhalis *strains and their isogenic *m35 *mutants used in this study are listed in Table 1. All strains were cultured at 37°C and 150-200 rpm in brain heart infusion (BHI) broth (Difco, Detroit, MI) or on BHI agar plates in an atmosphere containing 5% CO_2_. Media were supplemented with kanamycin (20 μg/ml) for culturing of the mutants. To investigate growth under different osmotic conditions, strains were cultured in BHI broth overnight at 37°C and 150 rpm. One ml of overnight culture was diluted 1:100 in fresh BHI supplemented with 0.25 M, 0.5 M or 1.0 M NaCl, respectively, and incubated at 37°C and 150 rpm. During cultivation to the stationary phase cell density was measured at OD_600_. The effect of exposure to different acidic environments was measured by growing bacteria in BHI broth overnight, harvesting and resuspending them in 20 mM Na_2_HPO_4_/NaH_2_PO_4_, 1 mM MgCl_2_, 25 mM L-arginine adjusted to pH 4.0, pH 5.0, pH 6.0, or pH 7.0, respectively. Suspensions were incubated for 2 h and 4 h, respectively, at 37°C, and the number of viable bacteria was quantified by plating of serial dilutions. Iron utilization experiments were performed by a disk feeding assay applying 5 μl of iron-saturated human lactoferrin (10 mg/ml) to sterile filter disks [30]. BHI agar plates were previously iron depleted by adding deferoxamine mesylate (Desferal, Novartis, Basel, Switzerland) to a final concentration of 30 μM and incubated at 4°C overnight before use. *Escherichia coli *DH5α was grown on Luria-Bertani (LB) agar plates or in LB broth.

**Table 1:** Bacterial strains used in this study

Strain	Description	Source or reference
*M. catarrhalis *O35E	middle ear isolate	[65]
*M. catarrhalis *O35E.*m35*	isogenic mutant strain, kan^R^	this study
*M. catarrhalis *300	nasopharyngeal isolate from child	[35]
*M. catarrhalis *300.*m35E*	isogenic mutant strain, kan^R^	this study
*M. catarrhalis *415	nasopharyngeal isolate from child	[35]
*M. catarrhalis *415.*m35* M. catarrhalis 287	isogenic mutant strain, kan^R^ nasopharyngeal isolate from child	this study [35]
*Escherichial coli *DH5α	Host strain for plasmid constructs	[66]

### DNA methods

Plasmids were isolated using the Wizard Plus SV Miniprep DNA purification system (Promega Corp., Madison, Wis.) DH5α was transformed as described previously [31]. Restriction enzymes were purchased from New England Biolabs, Inc., Beverly, MA. Electrocompetent *M. catarrhalis *was prepared and DNA was electroporated as described [32]. DNA sequencing was performed by using an ABI PRISM 310 genetic analyzer (PE Biosystems, Rotkreuz, Switzerland) with the Big Dye Terminator cycle sequencing ready reaction kit (PE Biosystems, Rotkreuz, Switzerland). Sequences were analyzed with the Lasergene software (DNASTAR Inc., Madison, WI). For sequencing of *m35 *of strain 287, DNA was amplified using the primers *m35*B5 (5'-TCGATACCAGAACACTACCTAAGC-3'), *m35*F2 (5' -GTCTGAGGGCAAGGTAGGCG-3'), *m35*RMJ1 (5' -CGTAGCAGTTTTCATCTCACCAC 3'), *m35*F3 (5'-CTTGCTCTAGCAACCGCAG-3'), *m35*R3 (5'-GCAAGACCTAGGTAAGTATC-3') and *m35*FMJ4 (5'-TGCGTGCATGGGTCGTGA-3').

### Construction of the isogenic mutants O35E.*m35*, 300.*m35 *and 415.*m35*

**Figure 1:**
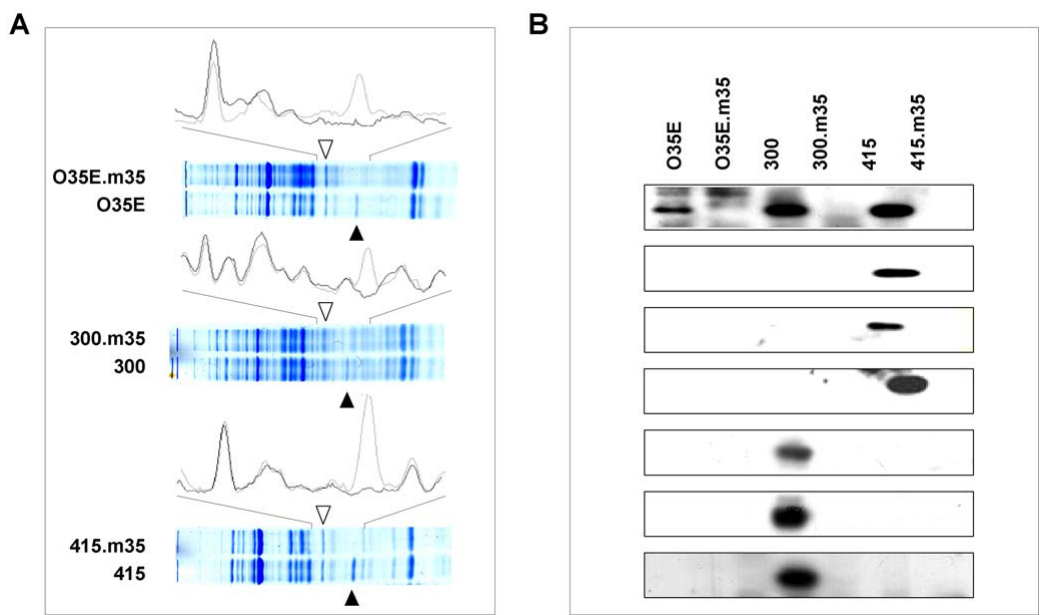
**(A) SDS PAGE of *M. catarrhalis *OMP of the strains O35E, 300 and 415 together with their respective *m35 *knock-out mutants**. Black triangles show the position of the M35 protein band at 36 kDa. White triangles show the position of the 40 kDa protein. The spectra display the intensity of each protein band determined by the AlphaEaseFC^® ^software. Light lines show the wild-type strain, dark lines show the respective M35 mutant strains. (B) Western Blot analysis for human salivary IgA against M35 of the strains O35E, 300 and 415 from seven healthy donors. The isogenic *m35 *mutants were included as negative controls.

Part of the *m35 *gene of the strains O35E, 300 and 415, respectively, was amplified using forward primer *m35*F3 (5'-CTTGCTCTAGCAACCGCAG-3') and reverse primer *m35*B5 (5'-TCGATACCAGAACACTACCTAAGC-3'). PCR products were ligated into the *BamH*I restriction site of pGEM-T-Easy pUC4K (Promega, Madison, USA). The kanamycin cassette was ligated into the *Avr*II restriction site of the *m35 *insert. The resulting construct, *Δm35:kan*, was used for electroporation of the competent strains O35E, 300 and 415, respectively. Transformants were selected on BHI agar plates containing 20 μg/ml of kanamycin. Insertional inactivation of *m35 *was confirmed by PCR analysis, sequencing, Southern blot analysis (data not shown) and immunoblotting (figure 1).

### Preparation of OMP

OMP were prepared by the EDTA buffer method as described [33]. Bacteria were harvested from a stationary phase culture, resuspended in EDTA buffer (0.05 M Na_2_HPO_4_, 0.15 M NaCl, 0.01 M EDTA, ph 7.4), homogenized and incubated at 55°C at 300 rpm for 1 h. Cells and cell debris were eliminated by centrifugation at 10,000 × g for 15 min at 4°C. Finally, OMP were collected by ultracentrifugation at 100.00 × g for 2 h at 4°C.

### 2D-Gel electrophoresis and MALDI-TOF

Analysis of M35 and other OMP spots of strain O35E was performed a described previously [34], except for the precipitation of the OMP, which was omitted.

### SDS-PAGE gel electrophoresis and immunoblot for detection of human anti-M35 IgA

Samples were resolved by SDS-PAGE using a 7.5% polyacrylamide gel. Band intensity was quantified using the AlphaEaseFC^® ^program from Inotech, Inc. Antibody detection was performed by Western blot analysis. Proteins were transferred to polyvinylidene difluoride (PVDF) membranes (Immobilon-P; Millipore Corp., Bedford, MA). IgA binding was detected using human saliva samples as primary antibody source and goat anti-human IgA, respectively, labeled with horseradish peroxidase (SIGMA) as secondary antibody. Super Signal West Pico Chemiluminescent Substrate (Pierce Chemical Co., Rockford, IL) was used for detection of antibody binding. Unstimulated human saliva was collected from healthy volunteers using Salivette sponges^® ^(Sarstedt, Nümbrecht, Germany), centrifuged for 5 minutes at 2000 rpm and stored at -20°C. All volunteers were laboratory researchers and provided oral informed consent. Sampling of saliva from healthy volunteers was approved by the local ethics committee.

### Antimicrobial resistance testing

The minimum inhibitory concentrations (MIC) of penicillin, ampicillin, amoxicillin, amoxicillin-clavulanate, cefuroxime, ceftriaxone, imipenem, meropenem, erythromycin, doxycycline, gentamicin, vancomycin, ciprofloxacin, levofloxacin, and moxifloxacin were determined by E-test^® ^(AB Biodisk, Sweden) according to the manufacturer's instructions.

### Autoagglutination and serum bactericidal assay

Overnight cultures were resuspended in PBS and adjusted to an OD_600 _of 2.0 in glass tubes. OD_600 _of the supernatants were determined after 15 and 60 minutes, respectively. Serum bactericidal assay were performed as previously reported [35].

### Human cell lines and growth conditions

Chang conjunctival cells and A549 lung cells were maintained in Eagle's minimal essential medium (Invitrogen, Basel, Switzerland) supplemented with 10% of heat-inactivated fetal calf serum, 100 units/ml penicillin, 100 μg/ml streptomycin, and 2 mM L-glutamine at 37°C in 5% CO_2_. The THP-1 human monocytic cell line was maintained in RPMI 1640 (Lonza, Basel, Switzerland) supplemented with 2 mM L-glutamine, 10% heat-inactivated fetal calf serum, 0.05 mM β-mercaptoethanol, 10 mM HEPES, 100 units/ml penicillin and 100 μg/ml streptomycin at 37°C in 5% CO_2_.

### Adherence and Invasion assay

The ability of *M. catarrhalis *to adhere to and invade human epithelial cells *in vitro *was measured as described previously [36]. Adherence and invasion was assessed on both Chang conjunctival cells and A549 lung cells as described [36,37]. Each strain was analyzed in triplicate in each experiment.

### Proinflammatory activity of M35 on human monocytes

The pro-inflammatory potential of *M. catarrhalis *OMP was described previously [38]. To investigate if M35 is an important mediator of proinflammatory cytokine release on the bacterial cell surface, THP-1 cells (1 × 10^6^/ml) were stimulated with different concentrations (1 × 10^5^/ml, 1 × 10^6^/ml, or 1 × 10^7^/ml) of heat inactivated strain O35E or the O35E.*m35 *mutant and incubated for 18 h at 37°C and 5% CO_2_. After incubation, cells were centrifuged for 2 min at 11,800 × g and supernatants were stored at -80°C. Cytokines were measured using the R&D Systems DY208 for human CXCL8/IL-8 and R&D Systems DY210 for human TNFα/TNFSF1A (R&D, Minneapolis, USA), respectively.

### Statistical analysis

Comparison of several test series was evaluated by analysis of variance (ANOVA). The significance of differences between treatment and control groups was determined using the two-tailed *t-*test. P < 0.05 was considered as statistically significant. Each value represents the mean ± one standard deviation of at least three independent experiments performed in triplicate.

## Results

### *In vitro *growth of m35 mutants

**Figure 2:**
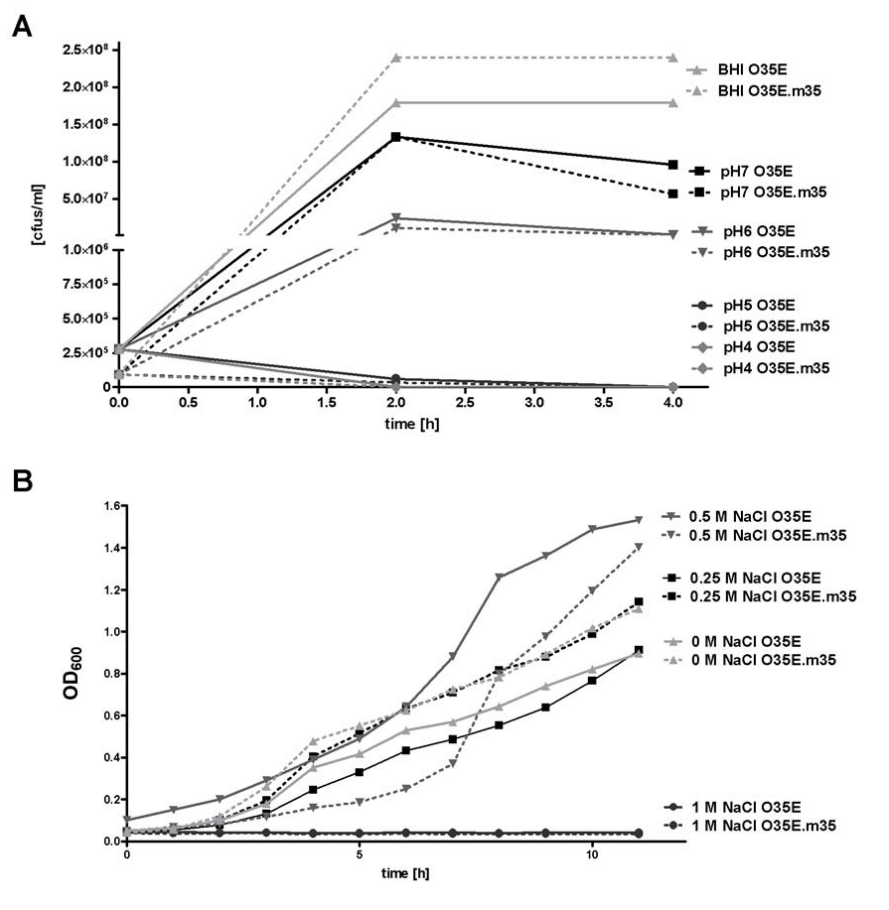
**Growth of *M. catarrhalis *O35E and its isogenic *m35 *mutant under acidic (A) and osmotic (B) stress**. (A) The effect of exposure to different acidic conditions was measured by growing bacteria in BHI adjusted to pH 4.0, pH 5.0, pH 6.0, or pH 7.0, respectively. Suspensions were incubated for 2 h and 4 h, respectively, and the number of viable bacteria was quantified by plating on BHI agar plates. (B) Different osmotic conditions were investigated by culturing bacteria in BHI supplemented with 0.25 M, 0.5 M or 1.0 M NaCl. During cultivation to the stationary phase bacterial density was measured at OD_600_.

Standard growth curves of the three wild-type/mutant pairs in BHI broth revealed no difference in growth velocity measured as broth density at OD_600 _(data not shown). Because porins are frequently involved in stress responses of bacteria against changes in osmolarity or pH [39-41], we investigated the wild-type/mutant pairs with respect to growth at various osmotic (supplementation of BHI with 0.25, 0.5 and 1 M NaCl) and acidic (pH 4-7) conditions (figure 2). Again, wild-type strains and their respective mutants behaved identically.

### *M35 *knockout is not associated with upregulation of a 40 kDa OMP

Easton et al. [6] described the upregulation of a 40 kDa protein in one of their isogenic *m35 *mutants. In order to confirm this observation, we compared Coomassie-blue stained OMP profiles of our three strains with their respective *m35 *mutants, but failed to detect any discernible upregulation of other OMP as determined by measuring protein band intensities (figure 1A). Thus, removal of M35 does not appear to affect the OMP composition when bacteria are grown in BHI. Taken together, the data presented thus far indicate that M35 is not essential for growth *in vitro *and that its removal from the OM does not otherwise affect the OMP composition.

### M35 is expressed *in vivo*

The human mucosal antibody response to OM components of *M. catarrhalis *has been described in detail [42-45], but M35 has never been paid specific attention. In order to search for human antibodies against M35, OMP of the strains O35E, 300, 415 and their isogenic mutants were resolved by SDS-PAGE, transferred to PVDF membranes, and incubated with human saliva for detection of IgA. Western Blot analysis for anti-M35 IgA with 7 different donors resulted in antibody signals to all three strains with the strains 300 and 415 displaying broader immunogenicity than O35E (figure 1B). Four of seven donor saliva contained anti-M35 IgA against strain 300 and 415, respectively. One donor displayed anti-M35 IgA for all three strains. OMP of the *m35 *mutants were used as negative controls (figure 1B). These data suggest that *M. catarrhalis *expresses M35 *in vivo *and that the mucosa-associated lymphoid tissue recognizes M35 as an antigen.

### Aminopenicillin susceptibility is mediated by M35

**Figure 3:**
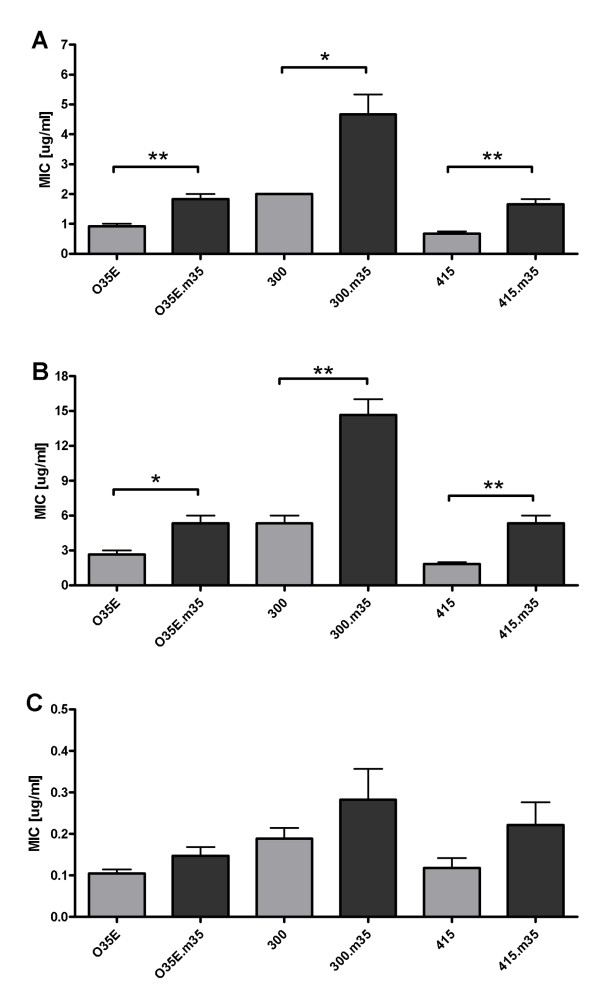
**E-tests of the strains O35E, 300 and 415 and their respective isogenic *m35 *mutants with ampicillin (A), amoxicillin (B) and amocixillin-clavulanate (C)**. Bacteria were cultured on agar plates together with E-test strips overnight. Bars show the minimum inhibitory concentration (MIC) for each antibiotic. Data are presented as means ± 1 SD (n = 3). * *p *< 0.05 for wild-type vs. respective mutant.

One of the major bacterial strategies for drug resistance is barrier protection, which limits the intracellular access of antimicrobial agents [18]. The influx of large, charged molecules is controlled by porins, which allow passive penetration of hydrophilic molecules of several classes of antibiotics [19-21]. Thus, porin-mediated OM permeability is expected to affect susceptibility to antimicrobial agents [18,22]. To investigate this, E-tests were performed with the mutants O35E.*m35*, 300.*m35*, 415.*m35 *and their respective wild-type parent strains. There were no differences in MIC for penicillin G, ceftriaxone, meropenem, erythromycin, doxycycline, gentamicin, and vancomycin between wild-type and mutant strains, respectively. For quinolones (ciprofloxacin, levofloxacin and moxifloxacin), cefuroxime and imipenem there was a minor, but consistent ~1.4-fold increase in the MIC of the mutants (data not shown). For ampicillin and amoxicillin, however, there was a statistically significant increase in the MIC of the mutants (2.5 to 2.9-fold) in comparison with their respective wild-types (figure 3A/B) (p = 0.003-0.023). Interestingly, at an approximately 10-fold lower level, this was also found for amoxicillin-clavulanate (figure 3C).

### Expression of M35 and putative virulence traits of *M. catarrhalis*

The capacity to autoagglutinate is mediated by hemagglutinin (also called *Moraxella *IgD-binding protein) [46], but some hemagglutinin knock-out mutants still autoagglutinate (unpublished data). Thus, we investigated whether the absence of M35 affected autoagglutination, but failed to identify any difference between strain O35E and O35E.*m35 *(data not shown). Similarly, resistance of *M. catarrhalis *to human complement, which is associated with disease-causing isolates [47-49] and which requires expression of several OMP [37,50-52], was not impaired by the lack of M35 (data not shown). Growth of *M. catarrhalis in vivo *is dependent on the ability to acquire iron from the human host by retrieving Fe^3+ ^from iron-containing host proteins by a number of specific binding and uptake systems [30,53-59]. Because of its abundance on mucosal surfaces, we chose to investigate the ability of the *m35 *mutant to use iron bound to human lactoferrin using a standard disk feeding assay on iron depleted BHI agar plates [30]. The experiment resulted in no differences in growth between the three *m35 *mutants and their respective wild-type parents (data not shown).

### Adherence and invasion of the *m35 *mutant

**Figure 4:**
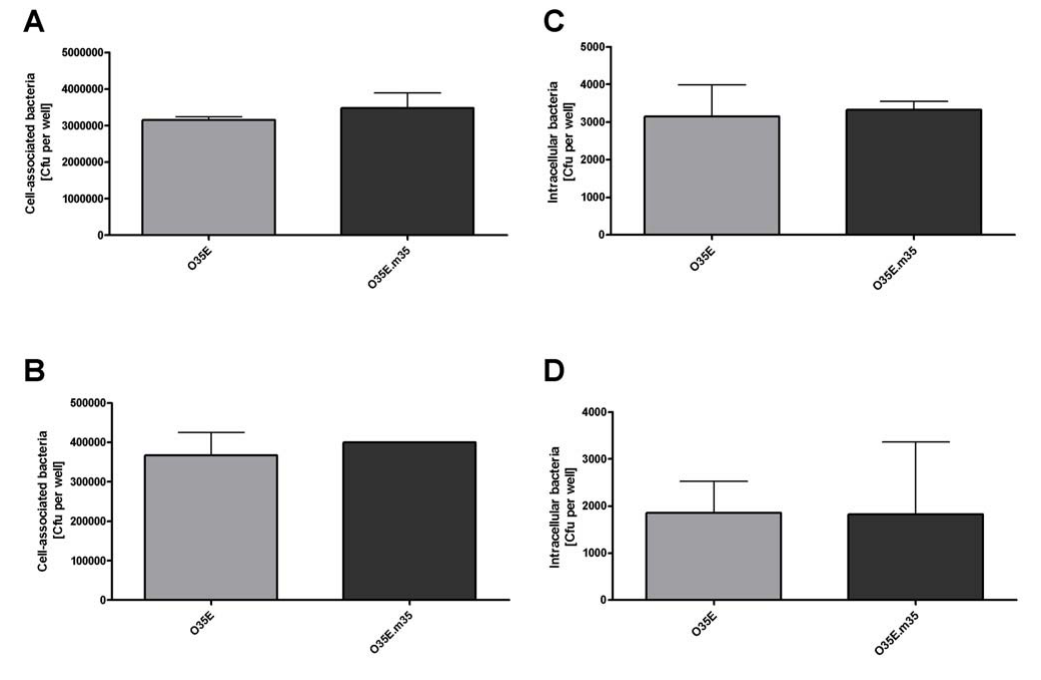
**Adherence to Chang conjunctival cells (A) and A549 lung cells (B) and invasion into Chang cells (C) and A549 cells (D) by *M. catarrhalis *O35E and its isogenic knock-out mutant O35E**. *m35*. The cells were infected and, after 30 min for adherence and 3 h for invasion, total cell-associated bacteria or intracellular bacteria, respectively, were quantitated by dilution plating. Data are represented as means ± 1 SD (n = 3) of at least three separate experiments.

The abilities of a pathogen to adhere to and invade epithelial host cells, respectively, are major virulence factors. Adhesins and invasins usually are OMP [5,36,60,61], some of which also act as porins [7-11]. To investigate if M35 mediates adherence and invasion assays were performed on Chang conjunctival cells as well as on A549 lung cells. Adherence of the O35E.*m35 *mutant was as efficient as that of its wild-type parent strain (figures 4A/B). Similarly, no differences were found for the capacity to invade these cell types (figures 4C/D) in gentamicin protection assays.

### Proinflammatory activity of M35 on human monocytes

Proinflammatory activity is typically induced by OMP, lipopolysaccharide or lipoteichoic acids. Porins have also been described to induce proinflammatory cascades by activating innate immune receptors mediating the expression of several chemokines and cytokines [11-17]. We investigated M35 with regard to its proinflammatory effect on human monocytes. THP-1 cells were stimulated with strain O35E or its O35E.*m35 *mutant overnight and cytokine release in the supernatant was measured by determining the concentrations of IL-8 and TNFα by ELISA. There was no difference in the release of IL-8 and TNFα, respectively, between wild-type and mutant (data not shown).

### *M35 *sequence analysis of type 2 strain 287

**Figure 5:**
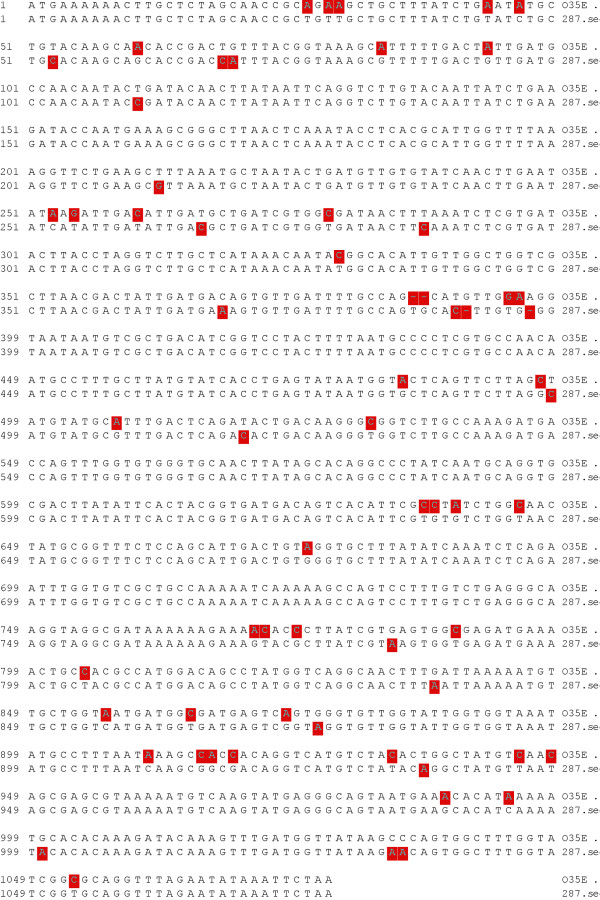
***M35 *gene DNA sequence of type 1 strain O35E compared with type 2 strain 287**. The red squares indicate diverse nucleotides between the two strains.

**Figure 6:**
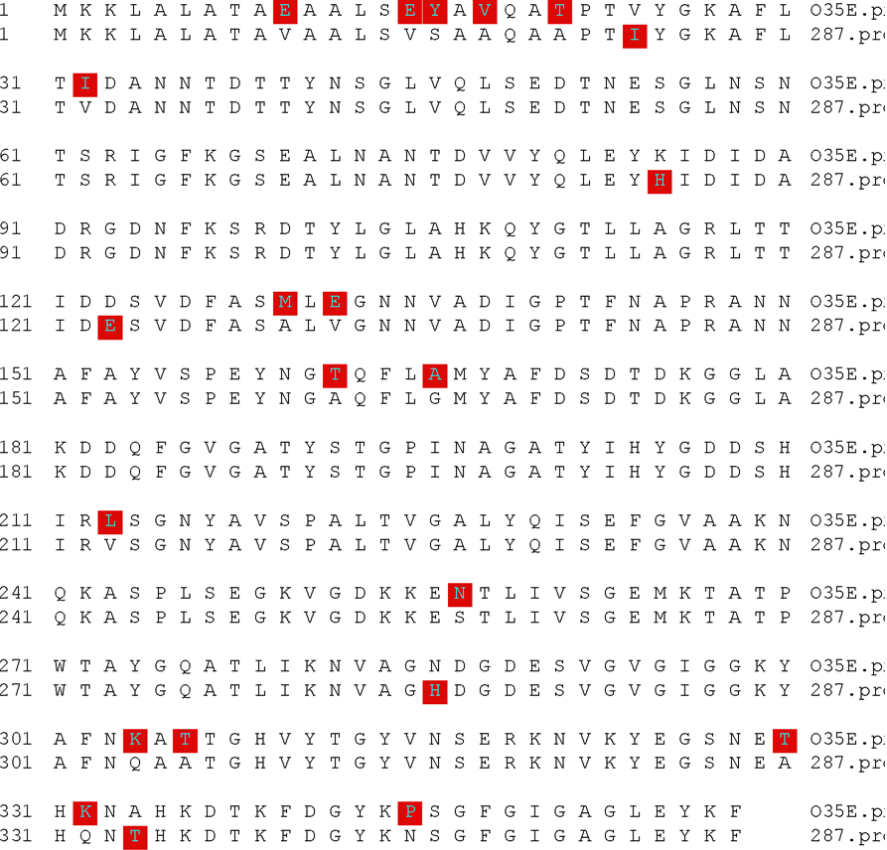
**Deduced M35 amino acid sequence of type 1 strain O35E compared with type 2 strain 287**. The red squares indicate diverse amino acids between the two strains.

*M35 *is nearly 100% conserved among type 1 strains of *M. catarrhalis *[28]. To determine if this is also true for the other major phylogenetic subpopulations of *M. catarrhalis, m35 *of type 2 strain 287 was sequenced and analyzed by bioinformatics. In comparison with type 1 strain O35E, there was a divergence of 5.8% at the DNA level consisting mainly of point mutations (figure 5). Unexpectedly, these DNA mutations lead to an even greater divergence of 6.1% at the amino acid sequence level (figure 6). This corresponds to a substantial number of missense mutations.

## Discussion

Porins are essential components of the gram-negative outer membrane and contribute to nutrient transport, antimicrobial resistance, response to osmostress and other processes, which are essential for bacterial homeostasis. M35 is the first functionally characterized OM porin of *M. catarrhalis *[28] and as of today all isolates examined carry a highly conserved *m35 *gene on their chromosome. This may indicate that strains not expressing M35 are not viable *in vivo*, but the only evidence in support of this hypothesis is the observation that a *m35 *mutant was unable to colonize the nasal mucosa of mice [6], which are not a natural host species for *M. catarrhalis*.

Our observation that healthy humans have mucosal IgA directed against M35 indicates that this protein is expressed *in vivo*. However, the fact that some salivary samples did not recognize all three M35 proteins tested suggests that either (i) antigenic variation occurs at immunoreactive, surface exposed epitopes, or (ii) it is a weak antigen and some individuals lose or never acquire anti-M35 IgA, or (iii) some isolates lack expression of M35 *in vivo*. If the latter were the case, screening of large collections of clinical *M. catarrhalis *isolates should identify strains either lacking a *m35 *gene or isolates carrying silent genes. The answer to this question is of clinical relevance, because our data indicate that the absence of M35 is a previously unknown mechanism of aminopenicillin resistance in *M. catarrhalis*. This effect could occur in *vivo *by alterations in porin expression to prevent antibiotic influx, which is a well known mechanism of resistance in other pathogens [18,22], and which often is associated with the expression of degradative enzymes to confer high level resistance [23,24]. The specificity of M35 for aminopenicillins could be explained by an effect Bezrukov et al described for OmpF of *E. coli*. They found that the nature and position of specific charges on both the antibiotic molecule and the porin play a major role in these interactions [25,26]. Strong binding to the pore correlates with high diffusion rates whereas weak binding is associated with reduced diffusion. As of today, the only known mechanism of aminopenicillin resistance in *M. catarrhalis *is the expression of one of three chromosomally encoded BRO betalactamases, which are detectable in greater than 90% of clinical isolates [62] and explain the fact that the MIC for amoxicillin-clavulanate in our experiments was at least 10-fold lower than the MIC for amoxicillin. This finding indicates that clavulanate penetrates the OM by ways other than M35. It also demonstrates that, in the presence of clavulanate, the reduced amount of aminopenicillins still penetrating the OM in *m35 *mutants suffices to inhibit growth. Thus, it appears that clavulanate inhibits essentially all betalactamase activity available and that naturally occurring isolates lacking M35 would not currently pose a substantial therapeutic problem in patients treated with betalactamase-resistant betalactams. However, standard dose or high dose amoxicillin still is the therapeutic standard for antimicrobial therapy of acute otitis media. Based on our data (figure 3), currently accepted pharmakinetic/pharmcodynamic (PK/PD) breakpoints for resistance against standard dose (≥ 1.0 μg/ml) or high dose amoxicillin (≥ 8 μg/ml), respectively, [63] thus predict that isolates lacking functional M35 may display clinically relevant aminopenicillin resistance. This is particularly relevant for the treatment of acute otitis media. Drug concentrations reached in the middle ear cavity are low in comparison with serum concentrations [64] and treatment failure is typically caused by insufficient drug concentrations in the middle ear fluid [64]. The list of betalactam antibiotics tested in this study is not exhaustive and it is conceivable that other drugs may also be affected by *m35 *mutations. Thus, further studies are needed to explore the potential impact of *m35 *mutants on antimicrobial treatment failures.

Multiple phenotypic tests that we carried out with three wild-type/mutant pairs failed to uncover an additional functions attributable to M35. None of these results is particularly surprising. The strength of these "negative" data lies precisely in the fact that we did not study one, but three different isolates and their respective mutants, which, taken together, provide firm evidence that M35 is not involved these various phenotypes in *vitro*. The analysis of three different wild-type/mutant pairs also lead to the conclusion that knocking-out *m35 *does not necessarily upregulate expression of a 40 kDa OMP as stated by Easton et al (figure 1A). Because these authors used a different strain, it is conceivable that they observed a strain-specific phenomenon, which does not represent the entire species. Finally, we sequenced *m35 *of a strain belonging to the phylogenetically old, second subpopulation of *M. catarrhalis *[29], which differs from the younger subpopulation by a considerably larger genetic diversity [29]. Indeed, we found a substantial number of sequence deviations, which, interestingly, were even greater at the amino acid level than at the DNA level. It is thus conceivable that type 2 strains exhibit functional and/or antigenic differences with respect to M35, which warrant further investigation.

## Conclusion

The significant increases in MIC for ampicillin and amoxicillin of the *m35 *mutants indicate that the OM porin M35 is involved in the uptake of aminopenicillins. This is a previously unknown mechanism of resistance in *M. catarrhalis*. It remains to be elucidated whether naturally occurring, disease causing strains of *M. catarrhalis *devoid of functional M35 exist, and whether they may contribute to clinical treatment failure. The fact that normal human saliva contains anti-IgA indicates that M35 is expressed *in vivo*, but that antigenic variation may be greater than previously appreciated. Thus, further studies are needed before M35 can be considered a potential vaccine candidate against *M. catarrhalis*.

## Abbreviations

ANOVA: analysis of variance; BHI: brain-heart infusion; COPD: chronic obstructive pulmonary disease; EDTA: ethylene-diamino-tetra-acetate; HEPES: Hydroxyethyl-1-piperazinyl-ethansulfonic acid; IL-8: interleukin-8; LB: Luria-Bertani medium; MALDI-TOF: matrix-assisted laser desorption/ionisation-time of flight; OD: optical density; OM: outer membrane; OMP: outer membrane protein; PBS: phosphate-buffered saline; PCR: polymerase chain reaction; PVDF: polyvinylidene difluoride; RPMI: Roswell Park Memorial Institute medium; SDS-PAGE: sodium dodecyl sulphate-polyacrylamide gel electrophoresis; TbpB: transferring-binding protein B; TNFα: tumor-necrosis factor α.

## Authors' contributions

MJ participated in conceiving the study, conducted the majority of the experimental work and drafted the manuscript. NH constructed the *m35 *mutants of the strains O35E, 300 and 415. VS participated in conceiving the study. RT performed and interpreted the comparative SDS-PAGE analyses of wild-type and mutant strains. AS performed and analysed the MALDI-TOF experiments. CA was the principal investigator, conceived the study and finalized the manuscript. All authors read and approved the final manuscript.
